# Magnesium, Iron, Zinc, Copper and Selenium Status in Attention-Deficit/Hyperactivity Disorder (ADHD)

**DOI:** 10.3390/molecules25194440

**Published:** 2020-09-27

**Authors:** Harry Robberecht, Annelies A. J. Verlaet, Annelies Breynaert, Tess De Bruyne, Nina Hermans

**Affiliations:** Laboratory of Nutrition and Functional Food Science, NatuRA (Natural Products and Food Research and Analysis), Department of Pharmaceutical Sciences, University of Antwerp, Universiteitsplein 1, B-2610 Wilrijk, Belgium; annelies.verlaet@uantwerpen.be (A.A.J.V.); annelies.breynaert@uantwerpen.be (A.B.); tess.debruyne@uantwerpen.be (T.D.B.); nina.hermans@uantwerpen.be (N.H.)

**Keywords:** ADHD, Mg, Fe, Zn, Cu, Se, elemental status

## Abstract

In this study, we critically review the literature concerning the relation of Mg, Fe, Zn, Cu and Se and attention-deficit/hyperactivity disorder (ADHD). Elemental status is estimated using peripheral blood parameters, hair, urine, daily intake and response to supplementation. The observed associations between concentration levels of the elements Mg, Fe, Zn, Cu and Se and ADHD symptoms are contradictory. This is partly due to the heterogeneity and complexity of the disorder. As a trend, lower ferritin and zinc levels can be observed. However, this correlation is not causative, as illustrated by placebo-controlled trials reporting conflicting evidence on the efficacy of supplementation. Well-defined studies on changes in concentration levels of the elements in relation to ADHD symptoms before and after treatment with therapeutics it will be possible to shed more light on the significance of these elements in this behavioral disorder. The discussion on whether a change in concentration of an element is cause or consequence of ADHD is not within the scope of this article.

## 1. Introduction

Attention-deficit/hyperactivity disorder (ADHD) is a neurodevelopmental disorder characterized by inappropriate levels of impulsive behavior, hyperactivity and/or inattention [1]. Although ADHD is a common disorder in childhood, adolescence and adulthood, this review focuses on childhood ADHD since the adult form shows other characteristics. Together with schizophrenia, autism and epilepsy, ADHD is one of the most-studied neurodevelopmental disorders. Depending on definition and study, this disorder is relatively common, affecting an estimated 5% to 12% of school-aged children [2]. Although numerous associations between potential etiological factors are published, such associations do not address causality and that not all are biologically relevant. Associations mentioned in the etiology and pathology of ADHD include genetic factors [3], interaction of genes and nutrition [4], epigenetic [5,6,7] and, environmental elements, together with stress. Nutrition and diet were also reviewed as influencing factors [8], as restriction and elimination diets have been tried in ADHD treatments [9]. Supplements [10], herbal and nutritional products (complementary medicine) [11] and their effects on the disorder have been thoroughly discussed. As potential causes for ADHD in children, oxidative stress [12], metal toxicity [13], decreased methylation of relevant genes [14], cerebral hypoperfusion [15] and mitochondrial dysfunctions [16] have been mentioned as potential causes. Gut microbiota have been recently associated with dietary patterns and linked to susceptibility to ADHD [17]. Dietary immunomodulation [18] and antioxidant treatment [19] have been discussed by our research group in some preview papers. An etiologic classification of ADHD [20] and dietary sensitivities for this disorder [21] have been reviewed.

In this paper, a literature search was performed on the status of Fe, Cu, Zn, Mg and Se and ADHD in children and adolescents, then reported observations were critically reviewed. Attention deficit disorder (ADD) also forms part of the ADHD spectrum; however, this search was limited to publications in which ADHD is explicitly mentioned. The review is limited to ADHD in children and adolescents. Hence, papers on the adult form of this disease were excluded [22,23] because of the possible interference of additional factors that may jeopardize conclusions and hamper comparison with children and adolescents (work, environmental pollution, alcohol abuse, smoking attitudes).

Literature was screened for publications between January 2010 and March 2020. Important older references mentioned in some papers are also included. We consulted PubMed, Science Direct, Web of Science and Google Scholar for the search. The terms ADHD, the various elements, elemental status, blood level and daily intake of Fe, Cu, Zn, Se and Mg were used as keywords in various combinations. Publications in other languages with an English summary were included. Plasma, or serum, was the principal monitored medium, but also red blood cells, hair, urine and daily intake were included to evaluate the elemental status. Although normal values for the various elements could be geographically different, the ADHD group was always compared with a control group for the same region. Concluding that there were deficiencies or excessive levels can only be done by using biological activity measurements of related proteins. Most of the time, the elements act as cofactors in various enzymes and proteins, and therefore could interfere in the etiology of ADHD. However, these proteins (studied in metallomes) were not included in this search strategy. Publications could mention only one element, or a combination with other elements could be presented.

The overall aim was to discuss found relations between the various parameters used for evaluating the elemental status and ADHD in children and adolescents and observed response on supplementation.

## 2. Magnesium (Mg)

Magnesium (Mg^2+^) is the second most abundant cation in the human intracellular compartment and is of great physiological importance [24]. It plays an important role in over 300 metabolic reactions involved in protein synthesis, nucleic acid production and cellular energy generation [25]. Therefore, low levels of magnesium have been associated with a number of chronic diseases. The role of magnesium in nerve tissue has been discussed [26]. Magnesium’s involvement in ADHD pathogenesis can originate from its role in the apoptosis of nerve cells by controlling the glutamate N-methyl-aspartate pathway [27] and its critical role in the conversion of essential fatty acids to omega-6 and omega-3 polyunsaturated fatty acids, which are important cofactors in the desaturase enzymes implicated in hyperactive behavior [28].

### 2.1. Magnesium Status

Magnesium deficiency is linked to disturbances in cognitive capability, leading to symptoms such as: fatigue, lack of concentration, nervousness, mood swings and aggression [29]. Since these symptoms are common in ADHD, it is not surprising that in most studies lower serum magnesium levels are reported for patients with ADHD compared to healthy subjects [30,31,32,33,34,35,36,37]. Nevertheless, in a minority of clinical studies ADHD patients have similar [38,39] or even higher serum levels than their healthy counterparts [28,40]. However, two recent reviews and meta-analyses on serum magnesium levels concluded that children with ADHD have lower levels than those without ADHD [41,42]. Four studies could be found, where lower erythrocyte magnesium levels [38,43,44,45] or lower Mg^2+^-ATPase activity have been identified [45]. Possibly intracellular magnesium is a better indicator of Mg status. Increased blood concentration, on the other hand could be due to therapeutic stimulant medications, as demonstrated [46]. Mg levels in saliva were significantly decreased in ADHD patients [47], as well as in hair [42,48]. Only one older publication could be found on magnesium in urine, reporting lower levels in ADHD subjects compared to healthy controls [30].

### 2.2. Magnesium Supplementation

A study in Australia showed a statistically significant association between higher dietary Mg intake and reduced externalizing behavior problems in adolescents [49]. Since a lower intake of magnesium in ADHD has been reported [50,51] many supplementation studies have been carried out to correct the magnesium homeostasis. Generally, these have been done in combination with omega-3 fatty acids, zinc and other minerals [29,45,52,53,54,55,56,57,58]. Some authors claimed that Mg supplementation may reduce ADHD symptoms in children with or at high risk of deficiency of this mineral [52,58]. However, systematic reviews [10,59,60,61] lead to the conclusion that evidence for efficacy of Mg supplementation in a non-Mg deficient ADHD population is lacking [10] and therefore cannot be recommended without demonstrated Mg deficiency in the treatment of ADHD [60].

## 3. Iron (Fe)

Iron is one the earliest described essential elements in humans. It is well studied in the field of hematology. Iron also plays an important role in basic brain function [62]. This element is a major cofactor of tyrosine hydroxylase, which is necessary for the synthesis of dopamine [63]. Therefore, iron deficiency may lead to a lower dopamine production and enhanced ADHD symptoms. Dopamine-receptor density and transport into the brain [64], but also a functional impairment in the dopamine-rich basal ganglia [65] can be important in the etiology of ADHD [66] Iron deficiency has been shown to affect cognitive motor, social and emotional functions in children [67] and is therefore thought to have a role in ADHD pathophysiology [68]. Hence, many studies have investigated the relationship between iron status and ADHD [68,69,70]. Various peripheral parameters on iron are considered: serum iron, serum transferrin, serum ferritin, hematocrit, hemoglobin, mean corpuscular volume (MCV), red blood cells (RBC) and total iron binding capacity (TIBC) levels. Quite recently, one publication on serum hepcidin levels could be found [71]. Furthermore, brain-iron and daily intake may be used as a measure of the mineral status, while the results on iron supplementation could be of some value in further evaluating the role of iron in ADHD.

### 3.1. Iron Status

#### 3.1.1. Peripheral Parameters

Literature data on the peripheral parameters of iron status in ADHD patients, compared to healthy controls and in relation to symptoms of ADHD is summarized in Table 1. Serum iron ferritin levels—considered to be a reliable indicator of body iron deposits—are mainly used to determine iron deficiency. Alternatively, serum iron levels or transferrin [72,73] have been mentioned, although results are controversial. Nevertheless, most of the studies indicate lower serum ferritin levels in ADHD patients [32,33,69,70,71,74,75,76,77,78,79,80,81,82,83,84,85,86,87,88,89,90,91,92,93,94]. Other reports found no significant relationship [89,95,96,97,98,99,100]. Only one (older) publication claimed to find higher levels [28]. In general, no differences in parameters related to anemia were observed compared to healthy controls: for serum iron [28,70,94,96], for transferrin, MCV [72] or whole blood [101]. To offer some explanation for these inconsistent results, perhaps research should focus on hepcidin, an important protein in iron metabolism and homeostasis of brain-iron [73]. The high levels of this protein found in ADHD patients may cause loss of iron homeostasis, but further studies are required to establish a definite conclusion [71].

#### 3.1.2. Hair and Urine Iron Levels

Iron levels in hair [30,102] and in urine [30] were found to be lower in patients compared to healthy controls.

#### 3.1.3. Brain Levels

Low thalamic iron level may play a role in the pathophysiology of ADHD. Three studies could be found on brain-iron levels in ADHD by using magnetic resonance imaging (MRI). In one of these studies Cortese et al. [85] found that thalamic iron levels in an ADHD group was significantly lower than the healthy controls. There was no significant correlation observed between the brain-iron levels and the level of ferritin in serum. Two other studies [103,104] compared brain-iron indices of medication-naïve ADHD patients with these of psychostimulant-treated ADHD patients and completely healthy controls. Iron analysis in the striatal and thalamic region of the medication-naïve ADHD patients revealed significantly lower levels than those in the other groups. Brain iron seems not only to be a noninvasive diagnostic biomarker that responds to treatment [103], it also seems that brain-iron levels in ADHD normalize as a function of treatment duration [104].

#### 3.1.4. Daily Intake

Most publications mention lower intake of Fe in ADHD children [55,56,105]. The treatment with methylphenidate may result in reduced appetite, and hence a lower intake of nearly all food components [55,56]. However, it was quite remarkable that the intake of overweight ADHD children was significantly lower compared to the normal weight ADHD group [106]. Authors do not offer any explanation for this observation. Only one older publication mentioned higher Fe intake in Taiwanese ADHD children, resulting in high blood-iron levels [107].

### 3.2. Iron Supplementation

Due to the low iron status found in most studies on ADHD patients, supplementation has been carried out with varying success. An iron preparation was mentioned as 80 mg/day [108] or 5 mg/kg/day [90]. Some of the studies reported that supplementation was effective in children with risk of iron deficiency and is well-tolerated [33,108,109,110]. Other researchers claimed that supplementation is effective especially in the inattentive subtype of the disorder [111], that it decreases risk of cardiovascular events during treatment with ADHD drugs [112]—or that it can be used as an intervention to optimize response to psychostimulants at a lower dose [91]. Sometimes a combination with Zn supplements was superior to iron alone in alleviating ADHD symptoms, as well as for improvement in performance in IQ tests [113]. The effect of iron supplements on iron blood parameters and on behavioral and cognitive symptoms in ADHD children without iron-deficiency merits further investigations [10,90]. After a systematic review of 11 randomized controlled trials, the authors claimed that more evidence was needed for an indication of an effect of iron (as well as magnesium and zinc) on the treatment of ADHD [61]. Indeed, iron overload (measured by higher serum ferritin levels) can become a risk factor for oxidative damage [90,111] and used as a marker of oxidative stress [114].

## 4. Zinc (Zn)

Zinc is an essential trace element, required for various cellular functions through proteins, enzymes and zinc-fingers [115]. Disturbance of Zn homeostasis is related to various disorders. Its role in metabolic syndrome and diabetic implications has been discussed in detail [116,117]. Enzymes involved in cell membrane stabilization or required for metabolism of neurotransmitters, melatonin [118] and prostaglandins are using zinc as cofactor. Dopamine transport is also regulated by this element [119,120]. In boys with ADHD the effects of zinc on information processing have been described [121].

### 4.1. Zinc Status

#### 4.1.1. Serum Zinc Levels

Case-control trials in several geographical areas have demonstrated lower zinc levels in children diagnosed with ADHD compared to healthy controls [30,32,33,55,82,101,121,122,123,124,125,126,127]. There has been discussion in literature on the lower values of zinc in children with ADHD [128], but without report of a range of normal levels clinical significance thereof is questionable. Only two publications could be found without difference in serum Zn levels [28,129]. Several meta-analyses suggest a significant association between low zinc levels and the diagnosis of ADHD [120,124,130,131]. An ongoing double-blind, placebo and active treatment controlled multicenter trial with ADHD children monitors the effect of Pycnogenol^R^ by measuring various parameters, including serum zinc concentrations [132].

#### 4.1.2. Hair and Urine Zinc Levels

Generally, lower zinc levels in hair were published in ADHD [30,48,102]. Isolated reports mentioned no difference [129] or even higher levels in hair of ADHD children [48]. However, the latter article would be more informative if this observation could be related to altered zinc levels in serum. Only one (older) publication could be found on zinc levels in urine of ADHD patients. Furthermore, here, patient baseline urinary zinc was significantly lower than in normal controls [133].

#### 4.1.3. Daily Intake

Nearly all studies found lower daily intake of zinc in ADHD children [49,50,55,56,125]. In only one publication zinc intake was not different in ADHD compared to healthy controls [28].

### 4.2. Zinc Supplementation

Although above mentioned observations suggest an association between low Zn status and ADHD, it has not been demonstrated that zinc deficiency is a causative factor for ADHD nor that zinc treatment is recommended, Zn supplementation studies have been carried out. Randomized, placebo-controlled trials of zinc supplementation either as an adjuvant to psychostimulant treatment or as monotherapy have provided conflicting evidence of efficacy [29,53,58,59,61,113,120,134,135,136,137,138,139,140,141,142]. Notwithstanding these large amount of studies, evidence is insufficient to recommend zinc supplementation, unless in areas with a high deficiency in Zn [143]. Furthermore, the dose and the form of zinc supplementation varied widely between the trials, so an optimal dosing strategy is not apparent [10].

## 5. Copper (Cu)

Copper is involved in catecholamine metabolism via beta-hydroxylase and monoaminoxidase (MAO). Higher copper concentrations lead to lower dopamine levels in rats [144]. In children, no correlation could be observed between whole blood Cu concentration levels and ADHD scores [101], nor was serum or plasma copper concentration different from that of normal controls [32,54,145]. In one publication a statistically insignificant higher (*p*  >  0.05) copper blood level, compared to this in the with ADHD was associated with a decreased serum Cu/Zn SOD (superoxide dismutase)-level [146]. Lower levels of copper, as found in an older publication [104], were confirmed by the group of Rucklidge [140,141]. However, the lower baseline Cu concentrations seem to have limited value for the identification of responders to treatment with a vitamin-mineral supplement, which was also demonstrated for a similar supplementation study on adults with ADHD [147]. The Cu/Zn ratio may be more important than the elemental concentrations separately, as Skalny and coworkers proved recently [127]. They claimed that an elevated Cu/Zn ratio may significantly contribute to the risk of ADHD or its severity and/or comorbidity. One publication mentioned a 19% reduction in the values of copper in hair of ADHD patients, compared to controls [48]. Analysis of food intake and nutrient status in children with ADHD shows lower Cu levels in children with a predisposition for ADHD [50]. No relation was observed between copper exposure during pregnancy and ADHD symptomatology at the age of 4 years [148]. As a consequence of these contradictory findings Cu supplementation studies have not been carried out or could not be found.

## 6. Selenium (Se)

Selenium (Se) is an essential element in a number of selenoproteins, including glutathione peroxidase (GPx), a family of enzymes that protects against oxidative injury by catalyzing the breakdown of hydrogen peroxides [149]. There are approximately 24 human genes encoding for selenoproteins [150]. Insufficient intake of Se results in increased risk of developing many chronic degenerative diseases. Keshan disease, for instance, is a typical example of endemic heart failure due to severe Se deficiency [151]. Therefore, it is believed that maintenance of Se status at a certain level is essential for human health. In most publications, assessment of Se status by measurement in serum or plasma, erythrocytes, platelets or whole blood has been reported. Some authors claim that a better assessment can be achieved by measuring the GPx-activity in whole blood or platelets. Serum or plasma reflects the recent daily intake, whereas erythrocytes accumulate Se and presumably reflect the intake over their 120-day lifespan [152]. Sometimes toenail [153] or hair [154,155] concentration is measured as a long-term parameter in Se status evaluation. Some biomarkers, such as the selenoproteins and particularly GPx 3 and SEPP1, directly provide information on biologic effect of the Se status and are of value in identifying nutritional Se deficiency and tracking responses of deficient individuals to Se treatment [156]. Since this trace element is an essential part of antioxidant enzymes, it can be linked to efficiency in lowering oxidative stress in ADHD. However, no significant differences in Se levels between controls and ADHD children could be found [126]. Low intake of Se was observed in ADHD groups compared to controls [55,56], although another study claimed to find no difference in Se intake [157]. Nevertheless, food rich in Se was inversely associated with ADHD [125]. Relatively higher umbilical cord Se level was observed for children, which afterwards manifested ADHD [158]. However, this single observation should be interpreted with caution.

## 7. Conclusions

The possible associations of the elemental status of Mg, Fe, Zn, Cu and Se with ADHD occurrence were reviewed. For some elements (Fe, measured as ferritin level and Mg), there is a trend of reporting lower values in the blood of ADHD patients. However, for both these elements, there were contradictory findings. This was also observed for other elements. These conflicting results mentioned in literature may depend largely on the amount of diagnostic parameters [159], treatments already received by patients, heterogeneity of the studies and variation in the daily intake of the various elements. Besides the limited number of studies carried out and found for each element, differences in the sampled population (gender, ethnicity, age, sample size) can jeopardize conclusions. Children and adolescents were not always in the same intervals of age. Other factors that were not mentioned in studies but were considered in the meta-analyses include stress, thyroid hormones and time at which the blood sample was taken. These variables may be important to consider in future research.

The discussion remains whether low status of various elements results from a decreased appetite as a consequence of ADHD medication. Another explanation of lower daily intake may be that patients with ADHD have an impaired ability to sit still during the meals, leave table earlier and have decreased nutritional intake for various nutrients. Therefore, daily intake should not be taken into account in assessing elemental status of the various elements. The discussion on whether a change in concentration of an element is cause or consequence of ADHD is not within the scope of this article. Evidence and arguments for supplementation of the various elements (alone or in combination) is insufficient, unless in a severe deficient status. Maybe, measurement of the nutrients or using peripheral parameters will evolve in the future into other directions. Research on biometals (metallomics), metalloproteins, metalloenzymes, chaperones (metallomes), as well as the application of genomics, proteomics and metabolomics, can contribute to an acceleration and improvement of identification of biomarkers in diagnosis and treatment of ADHD [160]. In this way new ADHD-biomarkers can be established and accepted [161].

## Figures and Tables

**Table 1:** Peripheral parameters of Fe status in attention-deficit/hyperactivity disorder (ADHD) patients compared to controls and relation with ADHD symptoms (“: the same as above; “-”: no additional remarks).

Parameter	Concentration Level	Observations/Remarks	Reference
Ferritin	Higher	-	[28]
	Similar	Low ferritin is related with sleep disturbances	[95]
	“	No relationship with symptoms	[96]
	“	No difference in ferritin level	[97]
	“	No difference in ferritin level	[98]
	Lower	-	[32]
	“	No causative relationship	[33]
	“	Link with ADHD and obesity	[69]
	“	Systematic review	[70]
	“	Contribution to ADHD	[74]
	“	No causative relationship	[75]
	“	Increased risk of restless legs	[76]
	“	Increased risk of restless legs	[77]
	“	Higher behavioral problems	[78]
	“	Related with behavioral, but not with cognitive problems	[79]
	“	Relation with sleep disturbances	[80]
	“	Inverse correlation with conners rating scale	[81]
	“	Higher hyperactivity	[82]
	“	After meta-analysis: higher susceptibility to ADHD	[83]
	“	Inverse correlation with conners rating scale	[84]
	“	Lower than in psychiatric controls	[85]
	“	Hyperactivity, reported by parents	[86]
	“	No relation with treatment outcome	[87]
	“	Associated with ADHD	[88]
	“	Correlated with hyperactivity scores	[89]
	“	Positive response on Fe supplementation	[90]
	“	Related to ADHD	[91]
Fe-serum	Similar	-	[70]
	Lower	-	[88]
Transferrin	Lower	-	[72]
Hepcidin	Higher	-	[71]
